# Machine learning-based CT texture analysis in the differentiation of testicular masses

**DOI:** 10.3389/fonc.2023.1284040

**Published:** 2024-01-16

**Authors:** Can Hu, Xiaomeng Qiao, Zhenyu Xu, Zhiyu Zhang, Xuefeng Zhang

**Affiliations:** ^1^ Department of Urology, The First Affiliated Hospital of Soochow University, Suzhou, Jiangsu, China; ^2^ Department of Urology, Suzhou Xiangcheng People’s Hospital, Suzhou, China; ^3^ Department of Radiology, The First Affiliated Hospital of Soochow University, Suzhou, Jiangsu, China; ^4^ Department of Urology, The Affiliated Hospital of Nanjing University of Traditional Chinese Medicine: Traditional Chinese Medicine Hospital of Kunshan, Kunshan, China

**Keywords:** contrast enhanced computerized tomography, CT texture analysis, testicular masses, machine learning, urology and radiology

## Abstract

**Purpose:**

To evaluate the ability of texture features for distinguishing between benign and malignant testicular masses, and furthermore, for identifying primary testicular lymphoma in malignant tumors and identifying seminoma in testicular germ cell tumors, respectively.

**Methods:**

We retrospectively collected 77 patients with an abdominal and pelvic enhanced computed tomography (CT) examination and a histopathologically confirmed testicular mass from a single center. The ROI of each mass was split into two parts by the largest cross-sectional slice and deemed to be two samples. After all processing steps, three-dimensional texture features were extracted from unenhanced and contrast-enhanced CT images. Excellent reproducibility of texture features was defined as intra-class correlation coefficient ≥0.8 (ICC ≥0.8). All the groups were balanced via the synthetic minority over-sampling technique (SMOTE) method. Dimension reduction was based on pearson correlation coefficient (PCC). Before model building, minimum-redundancy maximum-relevance (mRMR) selection and recursive feature elimination (RFE) were used for further feature selection. At last, three ML classifiers with the highest cross validation with 5-fold were selected: autoencoder (AE), support vector machine(SVM), linear discriminant analysis (LAD). Logistics regression (LR) and LR-LASSO were also constructed to compare with the ML classifiers.

**Results:**

985 texture features with ICC ≥0.8 were extracted for further feature selection process. With the highest AUC of 0.946 (P <0.01), logistics regression was proved to be the best model for the identification of benign or malignant testicular masses. Besides, LR also had the best performance in identifying primary testicular lymphoma in malignant testicular tumors and in identifying seminoma in testicular germ cell tumors, with the AUC of 0.982 (P <0.01) and 0.928 (P <0.01), respectively.

**Conclusion:**

Until now, this is the first study that applied CT texture analysis (CTTA) to assess the heterogeneity of testicular tumors. LR model based on CTTA might be a promising non-invasive tool for the diagnosis and differentiation of testicular masses. The accurate diagnosis of testicular masses would assist urologists in correct preoperative and perioperative decision making.

## Introduction

Testicular tumor is one of the most common malignancy in men aged 14-44 years worldwide, accounting for approximately 1% of all male tumors and 5% of genitourinary neoplasms. In recent years, the morbidity and mortality of testicular cancer has risen continuously, especially in Western countries (1–3). Testicular tumor is a heterogeneous group of diseases with various pathological subtypes and clinical behavior. Among them, 90%-95% are testicular germ cell tumors (TGCTs), including seminoma, embryoma, teratoma and choriocarcinoma, of which about 55% are seminoma of the testis. The other part of testicular tumor subtypes includes hematological neoplasm, sex cord stromal tumors, and other exceedingly rare types of tumors. As the different pathophysiology and molecular mechanisms, diverse biological behaviors were observed in these testicular masses, which leads to different management and clinical decision (4, 5). Of course, different treatment strategies are applied in benign or malignant testicular tumors and primary testicular lymphoma (6). Furthermore, as to these local or systemic progressed TGCTs, the main treatment is radiotherapy or chemotherapy instead of surgery (radical orchiectomy) (7). Under this circumstance, we cannot reach exact pathological results from the surgical specimens. Thus, a pre-operative diagnostic tool that allows histological subtype classification of testicular masses will be of great importance to precise treatment and clinical prognosis judgement. Although ultrasound examination is the preferred examination for testicular masses, the widespread use of ultrasound has led to more and more impalpable or ambiguous results (8). As mentioned by the EAU Guidelines 2022(http://uroweb.org/guidelines/compilations-of-all-guidelines/) (7), although magnetic resonance imaging (MRI) provides higher sensitivity and specificity than ultrasound in the diagnosis of testicular tumor, MRI is not superior to contrast enhanced computerized tomography (CECT) in detecting retroperitoneal lymph node metastasis in general and is more expensive, which does not justify its routine use in the diagnosis of testicular tumor (8, 9). Besides, it should only be considered when ultrasound is inconclusive, as local staging for testis-sparing surgery. However, CECT is recommended in all patients for staging before orchidectomy (7, 10). Therefore, CT has become an indispensable imaging method for patients with testicular masses. In addition, testicular biopsy is used in few centers and has not gained widespread acceptance because of narrow indication and possible increased local recurrence rate, with which it is difficult to assess intratumoral heterogeneity for its limitation (7, 11). In recent years, CT texture analysis (CTTA) has become a promising technique for evaluating tumor heterogeneity in a quantitative manner. CTTA could provide a measure of heterogeneity of testicular masses with various mathematical methods that can be used to evaluate the gray-level intensity and position of the pixels within contrast-enhanced CT images (12).

Up to now, no study has paid attention on the utility of CTTA in histological subtyping of testicular masses. This is the first study that explores the value of texture features in testicular masses.

## Materials and methods

### Patients

This study was approved by the Institutional Review Board in the First Affiliated Hospital of Soochow University with a waiver of informed consent. We retrospectively collected the imaging data and clinical data of consecutive 94 patients diagnosed with testicular masses from January 2015 to April 2022. Inclusion criteria were as follows: (a) patients with available three-phase CT scan prior to any treatment and operation; (b) pathologically proven testicular masses after surgery treatment; (c) the interval between CT and surgery was less than three months and no treatment received. Exclusion criteria included: (a) lack of pretreatment contrast-enhanced CT; (b) the absence of a certain phase of CT; (c) poor image quality. After conducting the criteria, 77 men were identified to constitute our study cohort and divided into a benign group (n=21) and a malignant group (n=56) according to their histological results. And then, in the malignant group, we divided them into primary testicular lymphomas group (n=10) and non-lymphomas group (n=46). Finally, we screened out all the testicular germ cell tumors from malignancy (n=43) and divided them into seminoma group (n=30) and non-seminoma group (n=13) for the differentiation.

### Study design

**Figure 1:**
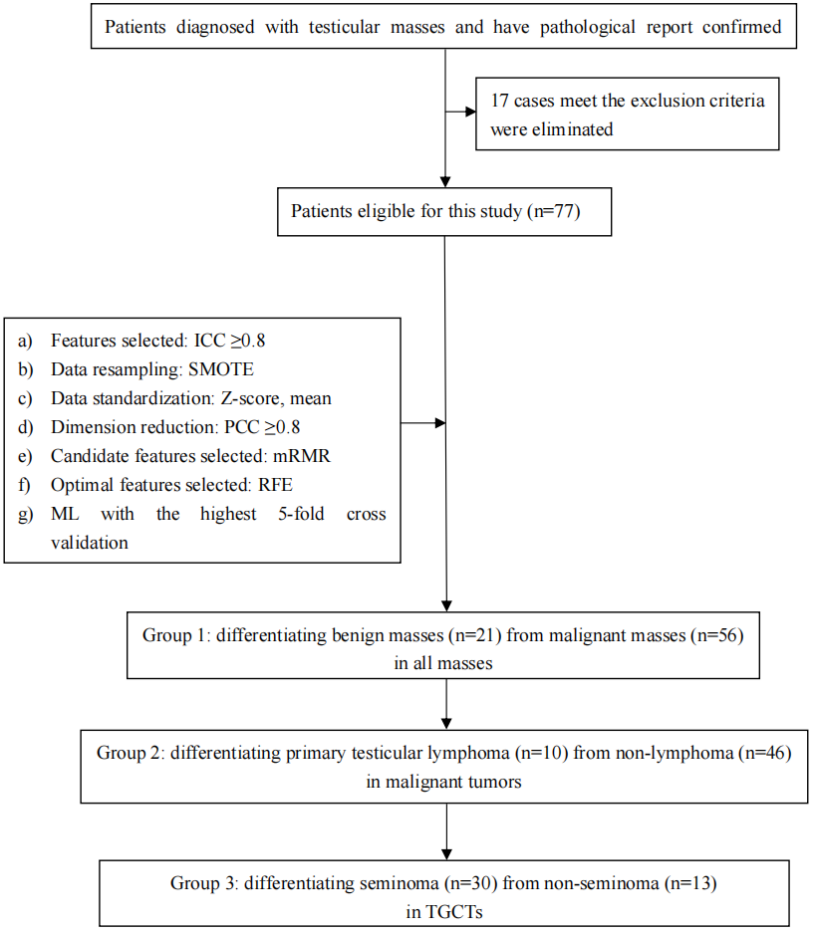
Simplified flow chart of the overall conceive of this study and the important steps in feature extraction, feature selection, and model optimization.

To make this article clear, a flow chart including specific technical steps was provided to the readers (**Figure 1**).

### Image acquisition

All patients underwent contrast-enhanced CT (GE Healthcare and Siemens Healthcare), including three phases: unenhanced phase (UP), arterial phase (AP, 9s delay after contrast injection) and portal venous phase (PP, 30s delay after contrast injection). Similar protocols were applied when scanning: tube voltage of 120 kVp, tube current of 180–450 mA, matrix of 512, field of view of 380–500 mm, and 5 mm reconstructed section thickness. Contrast medium (iopromide) was injected intravenously at a rate of 3.0 mL/s.

### ROIs delineation and data augmentation

**Figure 2:**
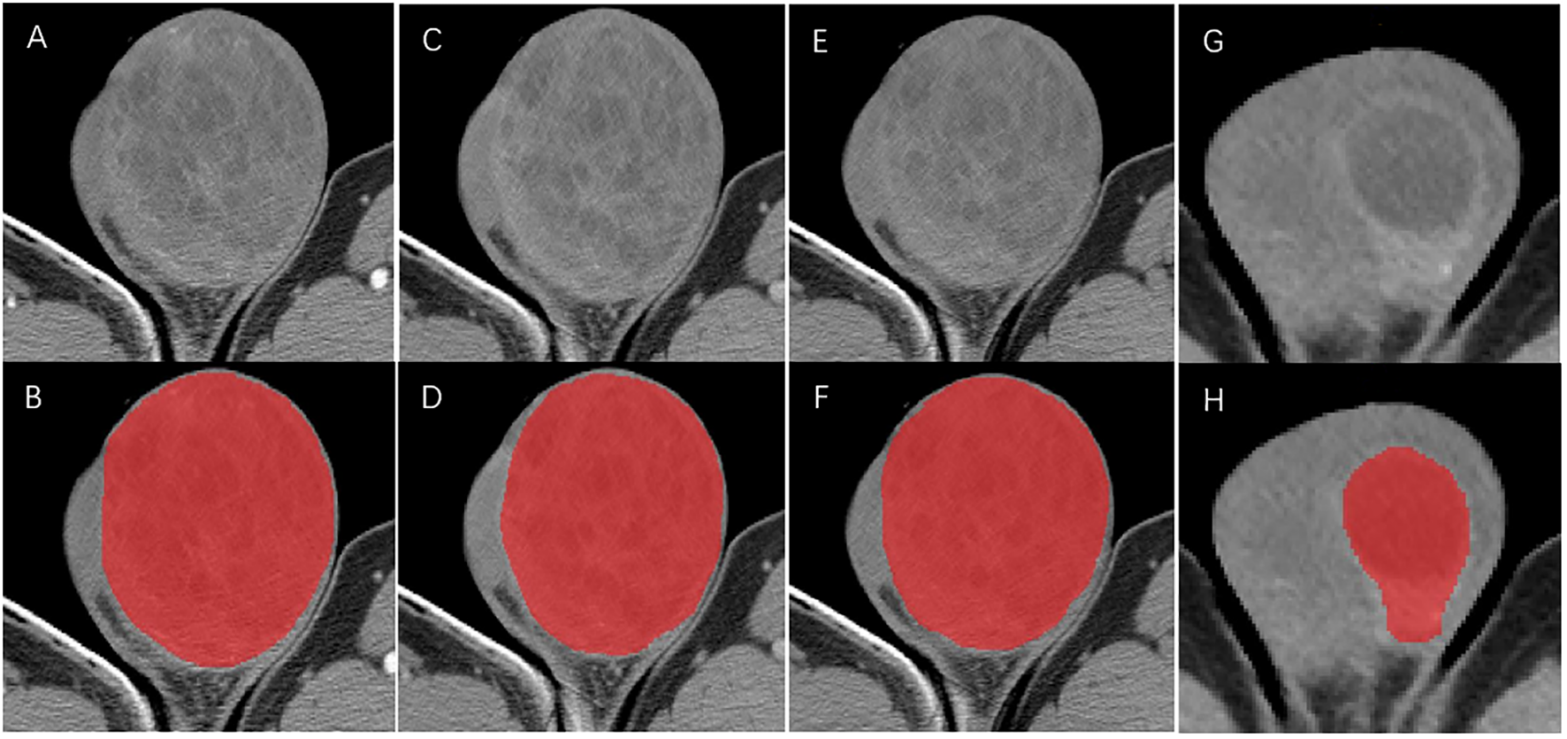
ROIs delineation in arterial phase **(A, B)**, portal venous phase **(C, D)** and unenhanced phase **(E, F)** CT for the mass without a distinct border. ROIs delineation in arterial phase **(G, H)** for mass with a distinct border.

For the mass without a distinct border or with invasion of the whole testicle, the region of interest (ROI) was defined as the whole testicular tissue on the diseased side. Meanwhile, for the mass with a distinct border, ROI was presumed to be the whole mass (**Figure 2**). One radiologist (with 5 years of experience) and one urologist (with 3 years of experience) blinded to the histopathology results first identified the border of each mass in consensus and then manually delineated the ROIs around the margin of the testicular masses with the ITK-SNAP (v 3.6.0) software (Can Hu and Xiaomeng Qiao). The ROIs were carefully drawn with an approximate distance of 1–3 mm from the margin of tumors to prevent the effect of fat and air (13). Due to the low morbidity of testicular tumors, sample size was inevitably limited in our study. Hence, as a scheme of data augmentation, the ROI of each patient was split into the upper and lower part by the largest slice and counted as two samples (for bilateral tumors, we counted one patient as four samples) (14). The histopathology results of augmented samples were in line with the original patients. After 2 weeks, the same task was repeated by the radiologist for the evaluation of intra-observer variation.

### CT texture feature evaluation and selection

Texture features were extracted from three-phases CT images. All CT images were anonymous before they were uploaded to the commercial texture analysis software (TexRAD, version 3.9, Feedback Medical Ltd) stored in DICOM format. A total of 572 features were extracted from each of the CT phases, including 18 first order features, 14 shape-based features, 24 features of grey level co-occurrence matrix (GLCM), 14 of grey level dependence matrix (GLDM), 16 of grey level run length matrix (GLRLM), 16 of grey level size zone matrix (GLSZM) and 5 of neighborhood grey tone difference matrix (NGTDM). The first order features and second order features were extracted from the original images and derived images via filtering based on the Laplacian of Gaussian. The spatial scale factor (SSF) at 6 levels (0 mm: no filtration; 2 mm: fine texture scale; 3 mm, 4 mm and 5 mm: medium texture scales; and 6 mm: coarse texture scale) were used. These features have been used in previous quantitative analysis studies and mathematical formula been described in the website in detail (https://www.ncbi.nlm.nih.gov/pmc/articles/PMC7581467/) (15–18).

Inter- and intra-observer intra-class correlation coefficient (ICC) was firstly utilized to assess reproducibility and repeatability for each texture feature. We retained features with ICCs greater than 0.8. A total of 985 texture features with ICCs ≥ 0.8 were included in the further feature selection process. In order to avoid the classifiers overtrained owing to highly-correlated features, feature selection dimension reduction was conducted to identify candidate and optimal features for model building (19). A synthetic minority oversampling technique (SMOTE) was adopted to deal with the adverse impact of the imbalanced data in this study. In addition, we also standardized the data by the method of Z-score and mean to compare the AUC of the model established by these two standardization methods for better model selecting. Dimension reduction was based on pearson correlation coefficient (PCC). Features demonstrating a strong correlation (PCC ≥0.8) were removed one by one to achieve better performance. Moreover, after the application of minimum-redundancy maximum-relevance (mRMR), each of the three groups for intra-group comparisons were reduced to 20 features. Before build the model, we also used recursive feature elimination (RFE) to further select optimal features with excellent discrimination ability from the above 20 texture features (20). Finally, with the highest 5-fold cross validation, five models were built by machine learning (ML) algorithms including auto encoder (AE), support vector machine (SVM), linear discriminant analysis (LAD), logistics regression (LR) and logistics regression-least absolute shrinkage and selection operator (LR-LASSO).

### Statistical analysis

Statistical analysis was performed using IBM SPSS v.23.0, Python software v2.7.13(https://www.python.org) and R software v.4.1.1. Non-normal distribution continuous variables were expressed as medians (interquartile range). The group differences were assessed using a Mann–Whitney U test. Receiver operating characteristic (ROC) curve analysis, accuracy, sensitivity, specificity, PPV and NPV were calculated to comprehensively assess the models. Significance between the AUC of models were compared using the Delong test. A two-sided p value <0.05 indicated statistical significance.

## Results

### Demographics

Specific pathological subtypes of all these testicular masses were provided in **Table 1**. Patient characteristics between the three groups were summarized in **Table 2**. Among them, 36 patients with lesions on the left side while 41 patients on the right side. Only one patient with granulosa cell tumor was bilateral. Thus, a total of 77 patients with 156 masses (76*2 + 1*4) were enrolled in the study according to our special method of data augmentation. For group 1, 21 benign cases and 56 malignant cases were counted. Statistical significance could be observed in age and all the serum tumor markers. For group 2, there were 10 primary testicular lymphomas (8 diffuse large B-cell lymphomas and 2 NK/T-cell lymphomas) and 46 non-lymphomas. The mean age of the lymphomas subgroup was statistically significantly higher than the non-lymphoma subgroup (33 (29, 39) vs 68 (58, 76), P <0.001). In the three serum tumor markers, LDH between the two subgroups had no significant difference. For group 3, there were 30 seminomas and 13 non-seminomas (9 mixed TGCTs, 2 embryonal carcinomas and 2 yolk sac tumors). Statistical significance could be observed in age, HCG and AFP. The average time interval between CT and serum tumor markers was 5 days.

**Table 1:** Specific pathological subtypes of testicular masses.

	Pathological subtypes	Specific subtypes	n
Malignant, n=56	TGCTs		43
		seminoma	30
		mixed TGCTs	9
		embryonal carcinomas	2
		yolk sac tumors	2
	Sex cord-stromal tumor		2
		granulosa cell tumor	1
		Sertoli-Leydig cell tumor	1
	Primary testicular lymphoma		10
		diffuse large B-cell lymphoma	8
		NK/T-cell lymphoma	2
	embryonal rhabdomyosarcoma		1
Benign, n=21		inflammation or abscess	5
		adenomatoid tumor	3
		angiomas or leiomyoma	8
		others	5

TGCTs, testicular germ cell tumors.

**Table 2:** Patients’ demographics between the three groups.

Variables, (M, IQR)	Benign vs Malignant	P	non-lymphomas vs lymphomas	P	non-seminoma vs seminoma	P
Age	47(35, 68) vs 35(29, 48)	0.041	33(29, 39) vs 68(58, 76)	<0.001	29(37, 34) vs 36(31, 43)	0.007
HCG	0(0, 0.2) vs 1.8(0.3, 38.8)	<0.001	3.8(1.0, 92.3) vs 0.18(0, 0.5)	<0.001	49.8(2.8, 246) vs 3.6(0.9, 38.8)	0.023
AFP	2.5(1.8, 3.1) vs 3.3(2.1, 18.3)	0.011	3.4(2.5, 112.2) vs 2.1(1.1, 5.0)	0.02	273(44, 381) vs 3.1(2.2, 4.2)	<0.001
LDH	173(151, 184) vs 219(172, 278)	<0.001	224(176, 290) vs 193(160, 270)	0.404	288(196, 327) vs 218(180, 251)	0.054

HCG, human chorionic gonadotropin; AFP, alpha fetoprotein; LDH, lactic dehydrogenase; IQR, interquartile range.

### Reproducibility and Feature selection

572 features were extracted from each of the CT phases. A total of 985 texture features with an ICC ≥0.8 were included in the further feature selection process. After mRMR, each of the three groups for intra-group comparisons were reduced to 20 features. Before model building, RFE was applied in all models to further select optimal features with excellent discrimination ability from the above 20 texture features (range from 9 to 15) (**Supplementary 1**).

### ML-based classifications

**Figure 3:**
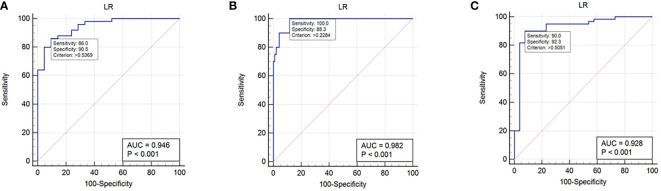
ROC curves in histological subtype classification of testicular masses using the selected logistics regression model. **(A)**: ROC curve for group 1. **(B)**: ROC curve for group 2. **(C)**: ROC curve for group 3.

The predictive performance and ROC curves of all ML and the two LR-based models using two data standardization methods for the three groups were summarized in **Tables 3A–C**, respectively. As a whole, z-score had a better performance than mean in the three groups. For group 1 (**Table 3A**), the LR and LR-LASSO were the two best-performing classifiers that achieved similar AUC values (AUC =0.946, P =1.000). However, considering the AUC of LR was slightly higher than LR-LASSO by the method of z-score, LR was selected for the best model. The overall accuracy, sensitivity, specificity, PPV, NPV and AUC of the best model were 87.3%, 86.1%, 90.5%, 95.6%, 73.1% and 0.946 (95% CI 0.896-0.995), respectively. For group 2 (**Table 3B**), although SVM and LR-LASSO had high AUC of 0.986 and 0.985, respectively, LR was chosen as the most appropriate model, achieved an accuracy of 90.4% (sensitivity 100%, specificity 88.3%, PPV 64.5% and NPV 100%) with an AUC of 0.982 (95% CI 0.963-1.000). For group 3 (**Table 3C**), LR also outperformed other models, achieving an accuracy of 90.7% (sensitivity 90.0%, specificity 92.3%, PPV 96.4% and NPV 80.0%) with a high AUC of 0.928 (95% CI 0.858-0.996). Overall, LR was the best choice for the histological classification of testicular masses. The ROC curves of LR among the three groups were demonstrated in **Figure 3**.

**Table 3A:** Performance of ML classifiers, LR and LR-LASSO in differentiating benign masses from malignant masses with the method of Z-score and mean.

Model	Standardization	ACC	SEN	SPE	PPV	NPV	Youden	AUC (95 % CI)
AE	mean	77.5%	74.0%	85.7%	92.5%	58.1%	0.59	0.833 (0.711-0.954)
	z-score	87.3%	92.3%	76.2%	90.2%	80.0%	0.68	0.866 (0.758-0.972)
SVM	mean	86.0%	84.1%	90.1%	95.4%	70.4%	0.74	0.922 (0.852-0.990)
	z-score	91.6%	94.2%	85.7%	94.0%	85.7%	0.80	0.900 (0.804-0.995)
LDA	mean	88.7%	87.9%	90.5%	95.7%	76.0%	0.78	0.910 (0.825-0.996)
	z-score	88.7%	88.3%	90.5%	95.7%	76.0%	0.79	0.910 (0.825-0.996)
LR	mean	87.3%	85.8%	90.5%	95.6%	73.1%	0.76	0.944 (0.892-0.995)
	z-score	87.3%	86.1%	90.5%	95.6%	73.1%	0.77	0.946 (0.896-0.995)
LR-LASSO	mean	88.7%	89.9%	85.7%	93.8%	78.2%	0.76	0.912 (0.836-0.988)
	z-score	88.7%	88.4%	90.5%	95.7%	75.0%	0.78	0.946 (0.894-0.996)

ML, machine learning; LR, logistics regression; LR-LASSO, logistics regression-least absolute shrinkage and selection operator; AE, autoencoder; SVM, support vector machine; LDA, linear discriminant analysis; ACC, accuracy; SEN, sensitivity; SPE, specificity; PPV, positive predictive value; NPV, negative predictive value; AUC, area under the curve; CI, confidence interval.

**Table 3B:** Performance of ML classifiers, LR and LR-LASSO in differentiating primary testicular lymphoma from non-lymphoma in malignant tumors with the method of Z-score and mean.

Model	Standardization	ACC	SEN	SPE	PPV	NPV	Youden	AUC (95 % CI)
AE	mean	93.7%	90.0%	94.7%	78.3%	97.8%	0.85	0.965 (0.930-1.000)
	z-score	87.6%	89.8%	86.2%	58.1%	97.6%	0.76	0.921 (0.847-0.995)
SVM	mean	93.7%	90.0%	94.7%	78.3%	97.8%	0.85	0.973 (0.947-0.998)
	z-score	93.0%	100%	91.5%	71.4%	100%	0.91	0.986 (0.970-1.000)
LDA	mean	87.7%	100%	85.1%	58.8%	100%	0.85	0.979 (0.957-1.000)
	z-score	87.7%	100%	85.1%	58.8%	100%	0.85	0.979 (0.957-1.000)
LR	mean	90.4%	100%	88.3%	64.5%	100%	0.88	0.982 (0.963-1.000)
	z-score	90.4%	100%	88.3%	64.5%	100%	0.88	0.982 (0.963-1.000)
LR-LASSO	mean	96.5%	85.0%	98.9%	94.4%	96.7%	0.84	0.978 (0.959-1.000)
	z-score	91.2%	100%	89.4%	66.7%	100%	0.89	0.985 (0.967-1.000)

ML, machine learning; LR, logistics regression; LR-LASSO, logistics regression-least absolute shrinkage and selection operator; AE, autoencoder; SVM, support vector machine; LDA, linear discriminant analysis; ACC, accuracy; SEN, sensitivity; SPE, specificity; PPV, positive predictive value; NPV, negative predictive value; AUC, area under the curve; CI, confidence interval.

**Table 3C:** Performance of ML classifiers, LR and LR-LASSO in differentiating seminoma from non-seminoma in TGCTs with the method of Z-score and mean.

Model	Standardization	ACC	SEN	SPE	PPV	NPV	Youden	AUC (95 % CI)
AE	mean	79.1%	85.0%	65.4%	85.0%	65.4%	0.50	0.765 (0.639-0.891)
	z-score	72.1%	75.0%	65.4%	83.3%	53.1%	0.40	0.673 (0.528-0.819)
SVM	mean	84.9%	83.3%	88.5%	94.3%	69.7%	0.72	0.890 (0.805-0.974)
	z-score	87.2%	83.3%	96.1%	98.0%	71.4%	0.79	0.919 (0.847-0.989)
LDA	mean	88.4%	91.7%	80.8%	91.7%	80.8%	0.72	0.912 (0.839-0.984)
	z-score	88.4%	91.7%	80.8%	91.7%	80.8%	0.72	0.912 (0.839-0.984)
LR	mean	90.7%	90.0%	92.3%	96.4%	80.0%	0.82	0.925 (0.858-0.996)
	z-score	90.7%	90.0%	92.3%	96.4%	80.0%	0.82	0.928 (0.855-0.994)
LR-LASSO	mean	86.1%	83.3%	92.3%	96.1%	70.6%	0.76	0.894 (0.808-0.978)
	z-score	86.2%	81.7%	96.2%	98.0%	69.4%	0.78	0.919 (0.848-0.988)

ML, machine learning; LR, logistics regression; LR-LASSO, logistics regression-least absolute shrinkage and selection operator; AE, autoencoder; SVM, support vector machine; LDA, linear discriminant analysis; ACC, accuracy; SEN, sensitivity; SPE, specificity; PPV, positive predictive value; NPV, negative predictive value; AUC, area under the curve; CI, confidence interval.

## Discussion

This is the first study that applied CT texture analysis (CTTA) to assess the pathological subtypes of testicular tumors. All the patients were divided into three groups to evaluate the ability of texture features for identifying benign and malignant testicular masses, identifying primary testicular lymphoma in malignant tumors and identifying seminoma in testicular germ cell tumors, respectively. For all three groups, the most appropriate model was LR rather than ML-based classifiers by the data standardization of z-score.

Testicular tumor is a heterogeneous group of diseases with various pathological subtypes and clinical behavior, which leads to different response to treatment (21). Firstly, the treatment of benign and malignant mass is different. Radical orchiectomy was the standard operation of TGCTs while symptomatic treatment is often used in benign masses. As to clinical stage I non-seminomas without vascular and lymphatic infiltration, retroperitoneal lymph node dissection (RPLND) is the standard treatment for patients without follow-up conditions. CTTA facilitates clinical evaluation and psychological development of patients, and to some extent RPLND could even be performed immediately after orchiectomy, avoiding the need for a second operation. As to clinical stage II TGCTs, seminomas tend to have sensitive response to radiotherapy while non-seminomas tend to benefit more from RPLND or neo-adjuvant chemotherapy. As to metastatic testicular tumors, urologists could only apply different chemotherapy regimens according to the prognosis (7, 22). Under the circumstance, exact pathological results cannot be reached from the surgical specimens. Conventionally, ultrasound examination is the preferred choice for testicular masses. Despite its high sensitivity in the mass detection, it shows low specificity in distinguishing between benign and malignant masses, let alone other pathological subtypes (23, 24). Furthermore, testicular biopsy is used in some centers but has not gained widespread acceptance because of narrow indications and concerns for tumor seeding along the biopsy tract. Germ cell neoplasia *in situ* (GCNIS) could be diagnosed by testicular biopsy using immunohistochemistry with high sensitivity and specificity. However, a certain amount of false-negative biopsy was brought inevitably (25). Thus, non-invasive test for the evaluation of testicular masses may open the possibility of allowing histological subtype classification.

CT is recommended for the pre-surgical assessment of testicular masses, and at the same time, could evaluate retroperitoneal lymph node metastases. However, the heterogeneity of tumors is not particularly obvious on imaging and the diagnostic accuracy depends on the experience of radiologists. In the present study, we found that quantitative CTTA potentially allowed for detection of subtle differences and was able to differentiate various histological subtype classifications beyond visual assessment. To date, as far as we know, there have been no CTTA related studies on testicular tumors. Previous research has focused on tumors such as epithelial ovarian carcinoma, renal cell carcinoma or lung carcinoma (26–29). In the study of An et al. (26), they demonstrated that CTTA was instrumental in the identification of high-grade serous carcinoma (HGSC) or non-HGSC in 205 patients. Erdim et al. (28) investigated that renal masses with unclear pathological diagnosis could be distinguished through ML-based CTTA in 79 patients. Furthermore, Ceyda et al. (27) has confirmed the ability of different ML-based classifiers in the prediction of Fuhrman nuclear grade of clear cell renal cell carcinomas in 53 patients. Yang et al. (29) evaluated the value of 2D and 3D CTTA in predicting lymphatic vascular invasion in lung adenocarcinoma.

Our study is not only focused on the differentiation of benign and malignant lesions but also on identifying primary testicular lymphoma in malignant tumors and identifying seminoma in TGCTs. The differential diagnosis of TGCTs or non-TGCTs was not included in our study for the reason that most testicular tumors were germ cell neoplasms (accounting for 95%), and the remaining few were of no great discriminative value and had a low incidence. To avoid confounding bias, we also did not identify lymphoma and seminoma across all tumor types. We think the above process may be more appropriate and in line with the clinical practice. The performance of most classifiers in all three groups are satisfactory. Despite the ACC of SVM is slightly higher than LR in group 1, we chose LR as the best classifier for the better stability of the model (the AUC of LR was higher than SVM) (30). For group 2, SVM and LR-LASSO seem to outperform LR (P >0.05). Nevertheless, compared to LR, the AUC of the two classifiers had a relatively large reduction when using the data standardization of mean. For group 3, LR was obviously superior than other models (P <0.05). Therefore, in view of the fact that the diagnostic performance of each model was not significantly different, we still tend to choose LR as the last model for uniformity. In general, CTTA could be potentially valuable in guiding treatment and provide a reliable reference for clinicians.

The result of optimal features indicated that the entropy of the gray-level cooccurrence matrix (GLCM) for AP, energy of the first-order texture feature for PP and 90^th^ percentile of the first-order texture feature for UP were features with the largest coefficient for the three groups, respectively. For group 1, malignant testicular tumors were characterized by a greater entropy for AP (P =0.028). Entropy represents the randomness or complexity of the texture in the image and a greater entropy tends to reflect heterogeneity, which exactly demonstrated the invasive growth pattern with poorly defined boundaries in malignant tumors (31–33). In addition, malignant testicular tumors appear to be more irregular on cells for the different degree of the disturbed formation of the germ cells (22, 34). Energy is the sum of the squares of voxel values and reflects the uniformity of image gray distribution and texture thickness (35, 36). Primary testicular lymphoma displayed a lower energy (P <0.001) and it may be associated with a worse overall survival and more aggressive tumors (36, 37). We also found that higher 90^th^ percentile was correlated with seminomas (P =0.020), demonstrating a phenomenon of hyper-attenuation in UP (38). Possible explanation for this is that seminomas typically have homogenous internal attenuation while non-seminomas show inhomogeneous soft-tissue density (39). Moreover, as the representation of low attenuation, hemorrhage and necrosis of seminomas may present but are usually limited (40, 41).

There are several limitations in our study. First, owing to the low morbidity, the sample size of the study is small inevitably. We had to apply the method of data augmentation to expand the sample size, which may aggravate selection bias. Secondly, no comparison was made with MRI and ultrasound in terms of diagnostic efficacy because not all patients had complete imageological examinations. Besides, as a comparative analysis with CTTA with other experimental methods like flow cytometry, H&E, IHC that would help to accurately diagnose the tumors based on CTTA. We look forward to further research on MRI and detecting techniques in the identification of testicular tumors. Thirdly, the potential impact of this methodical difference on clinical findings is largely unexplored. the reproducibility of texture analysis has yet to be established widely. Some issues like image acquisition and image quality, and their effect on texture analysis need to be regulated and resolved. Fourthly, our study was retrospective and lack of external validation. Although 5-fold cross validation was used, the risk of overfitting could not be avoided. Fifthly, a three- dimensional CTTA may be time-consuming, but this exactly the advantage of our study. Lastly, we chose only a few representative ML classifiers. Lastly, different devices and software may have different consequences. Thus, large-scale and well-designed studies are warranted to validate the performance of the models.

## Conclusion

In conclusion, LR model based on CTTA might be a promising non-invasive tool for the diagnosis and differentiation of testicular masses. The accurate diagnosis of testicular masses would assist urologists in correct preoperative and perioperative decision making.

## Data availability statement

The original contributions presented in the study are included in the article/**Supplementary Material**, further inquiries can be directed to the corresponding author/s.

## Ethics statement

This study was approved by the Institutional Review Board in the First Affiliated Hospital of Soochow University. The studies were conducted in accordance with the local legislation and institutional requirements. The participants provided their written informed consent to participate in this study. Written informed consent was obtained from the individual(s) for the publication of any potentially identifiable images or data included in this article.

## Author contributions

CH: Data curation, Formal analysis, Investigation, Resources, Writing – original draft, Writing – review & editing. XQ: Data curation, Formal analysis, Methodology, Software, Writing – original draft. ZX: Data curation, Formal analysis, Investigation, Writing – original draft. ZZ: Project administration, Supervision, Validation, Visualization, Writing – review & editing, Resources. XZ: Conceptualization, Funding acquisition, Investigation, Project administration, Supervision, Validation, Visualization, Writing – review & editing.
